# Relationship between Glycated Hemoglobin (HbA1c) in Adolescents with Type 1 Diabetes Mellitus (T1DM) and Parental Anxiety and Depression

**DOI:** 10.1192/j.eurpsy.2023.455

**Published:** 2023-07-19

**Authors:** E. Silina, M. Taube, M. Zolovs

**Affiliations:** ^1^Doctoral studies, Riga Stradins University, Riga; ^2^The Seaside Hospital, Liepaja; ^3^Riga Stradins University, Department of Psychiatry and Narcology; ^4^Statistics Unit, Riga Stradins University, Riga; ^5^Institute of Life Sciences and Technology, Daugavpils University, Daugavpils, Latvia

## Abstract

**Introduction:**

T1D is the most common chronic endocrine pathology in children. The management of type 1 diabetes requires strong diet, physical activity, lifelong insulin therapy, and proper self-monitoring of blood glucose and is usually complicated and, therefore may result in a psychosocial problem for the whole family. Metabolic control of the disease is determined by glycated haemoglobin (HbA1c), the main criterion for diabetes compensation. It is assumed that anxiety and depression symptoms negatively affect glycemic control. A correlation was observed between anxiety and depression level and glycaemic control, as well as a three-way interaction among HbA1c, frequency of blood glucose monitoring, and diabetes-related stress (Buchberger et al., 2016). Parental psychological distress was associated with higher child self-report of stress and depressive symptoms, and it had negative effects on diabetes management.

**Objectives:**

To evaluate the relationship between parental depression and anxiety and metabolic control of their adolescents with T1DM.

**Methods:**

The cross-sectional study recruited adolescents with T1D (N=251) and their parents (N=251). The 7-item Generalized Anxiety Disorder (GAD-7) scale measured anxiety level. The Patient Health Questionnaire – 9 (PHQ-9) detected depressive symptoms. Glycaemic control of patients was assessed using the last glycated haemoglobin (HbA1c) values. GLM mediation analysis was performed to determine the potential mediating effect of parent’ mental health depression and anxiety on the relationship between depression and anxiety of child on the level of HbA1c.

**Results:**

502 respondents were eligible for screening. Mediation analysis was performed to assess the mediating role of parent GAD-7 on the linkage between HbA1c and child GAD-7 and child PHQ-9. the results relevated that the total effect of child GAD-7 on HbA1c was significant but the total effect of child PHQ-9 was not significant. With the inclusion of the mediating variable (parent GAD-7) (Figure 1), the impact of child GAD-7 and child PHQ-9 was founding insignificant (p ≥ 0.05) but the indirect effect of child GAD-7 and child PHQ-9 on HbA1c though parent GAD-7 was found significant (p ≤ 0,01) (Table 1). This indicates that the relationship between HbA1c and child GAD-7 and PHQ-9 is fully mediated by parent GAD-7.

**Image:**

**Figure:**
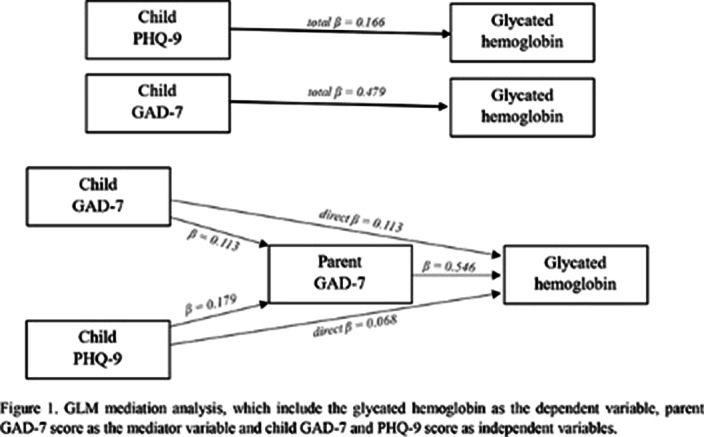

**Image 2:**

**Figure:**
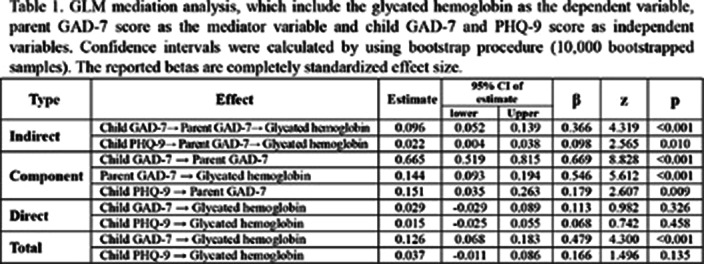

**Conclusions:**

Glycated haemoglobin in adolescents with Type 1 diabetes is related to adolescents’ mental health via parents’ anxiety. It means that parents’ anxiety plays more significant role in the level of glycated haemoglobin in adolescents than depression and anxiety of the adolescent.

**Disclosure of Interest:**

None Declared

